# Supplementary material for: High-resolution structure and biochemical properties of the LH1–RC photocomplex from the model purple sulfur bacterium, Allochromatium vinosum
Source: Commun Biol. 2024 Feb 12;7:176. doi: 10.1038/s42003-024-05863-w (PMC10861460; doi:10.1038/s42003-024-05863-w)
Supplement: Supplementary file 2 — Supplementary information [file 42003_2024_5863_MOESM2_ESM.pdf]

SUPPLEMENTARY INFORMATION

High-Resolution Structure and Biochemical Properties of the LH1–RC Photocomplex from the Model Purple Sulfur Bacterium, *Allochromatium vinosum*

K. Tani, R. Kanno, A. Harada, et al.

Supplementary Table 1 Comparison of the distances of His–BChl(Mg) and BChl(Mg)–BChl(Mg) in LH1 and RC special pairs from various phototrophic bacteria.

	Distance of His(Nε2) to BChl–Mg (Å)		Distance of Mg–Mg (Å)		
**LH1** (dimeric BChl)	α	β	Long	Short	PDB ID
Alc. vinosum	**2.68**	**2.21**	**9.34**	**8.23**	**8WDU**
Rba. capsulatus	2.63	2.23	9.46	8.28	7YML
Alc. tepidum	2.54	2.29	9.52	8.75	7VRJ
Rpi. globiformis	2.41	2.08	9.40	8.58	7XXF
Rba. sphaeroides (Dimer)	2.58	2.21	9.45	8.36	7VY2
Rba. sphaeroides (Monomer)	2.58	2.20	9.55	8.37	7F0L
Rsp. rubrum	2.27	2.03	9.34	8.51	7EQD
Rps. palustris (W)	2.93	2.71	9.61	8.2	6Z5S
Tch. tepidum	2.19	2.19	8.88	8.72	5Y5S
Trv. strain 970	2.60	2.32	8.90	8.47	7C9R
Blc. viridis	2.54	2.25	8.8	8.5	6ET5
Rfx. castenholzii (B880)	2.32	2.29	9.5	9.3	5YQ7

**RC** (special pair)	L-subunit	M-subunit	BChl *a* (L)–BChl *a* (M)	
Alc. vinosum	**2.08**	**2.26**	**7.65**	**8WDU**
Rba. capsulatus	2.28	2.33	7.65	7YML
Alc. tepidum	2.44	2.49	7.97	7VRJ
Rpi. globiformis	2.25	2.24	7.73	7XXF
Rba. sphaeroides (LH1–RC)	2.22	2.13	7.85	7VY2
Rba. sphaeroides (RC-only)	2.27	2.06	7.84	2J8C
Rsp. rubrum	2.09	2.12	7.76	7EQD
Rps. palustris	2.73	2.74	7.69	6Z5S
Tch. tepidum	2.17	2.19	7.87	5Y5S
Trv. strain 970	2.32	2.31	7.62	7C9R
Blc. viridis	2.36	2.35	7.83	6ET5

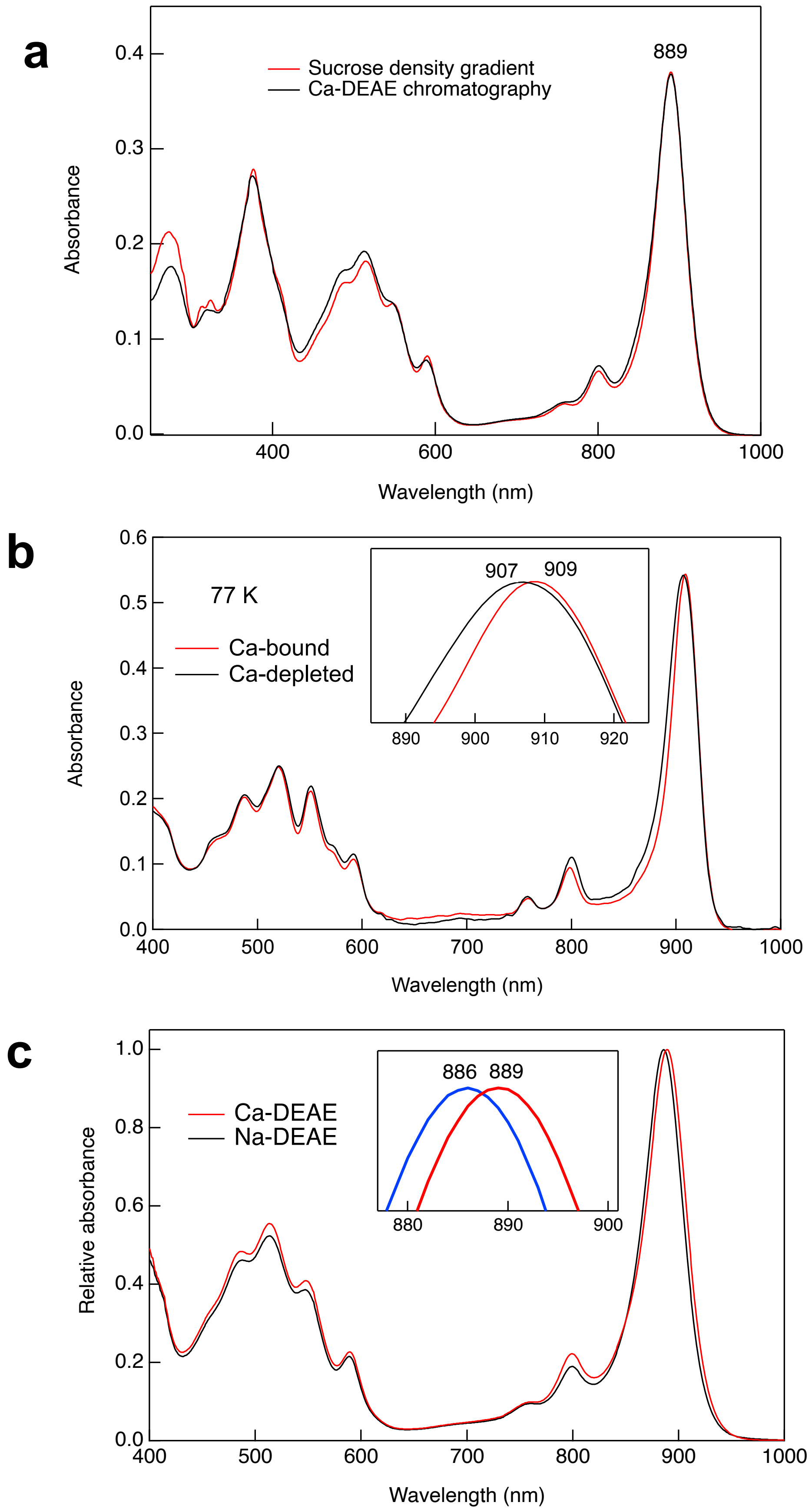

Supplementary Fig. 1 Absorption spectra of the *Alc. vinosum* LH1–RC complex. (a) Absorption spectra of the LH1–RCs purified by sucrose density gradient centrifugation and Ca-DEAE chromatography. **(b)** Absorption spectra at 77 K for the same LH1–RC samples used in Fig. 4a. **(c)** Absorption spectra of the LH1–RCs purified by Ca-DEAE and Na-DEAE chromatographies.

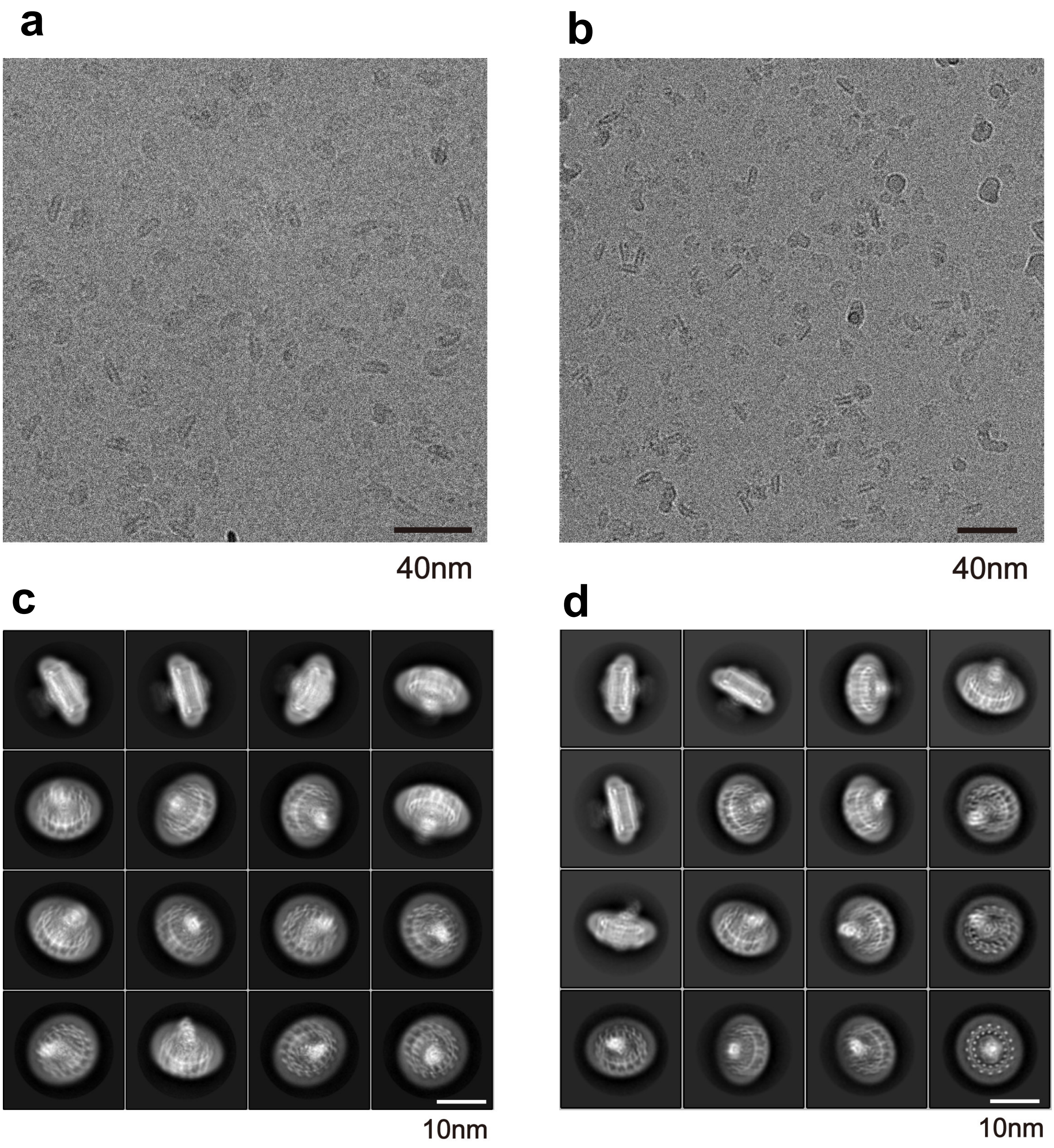

Supplementary Fig. 2 Electron micrographs of the *Alc. vinosum* LH1–RC complex. Representative cryo-EM (**a, b**) micrographs of the sucrose-density and Ca-DEAE purified LH1–RC samples, respectively. (**c, d**) Representative 2D class averages from cryo-EM micrographs of the sucrose-density and Ca-DEAE purified LH1–RC samples, respectively.

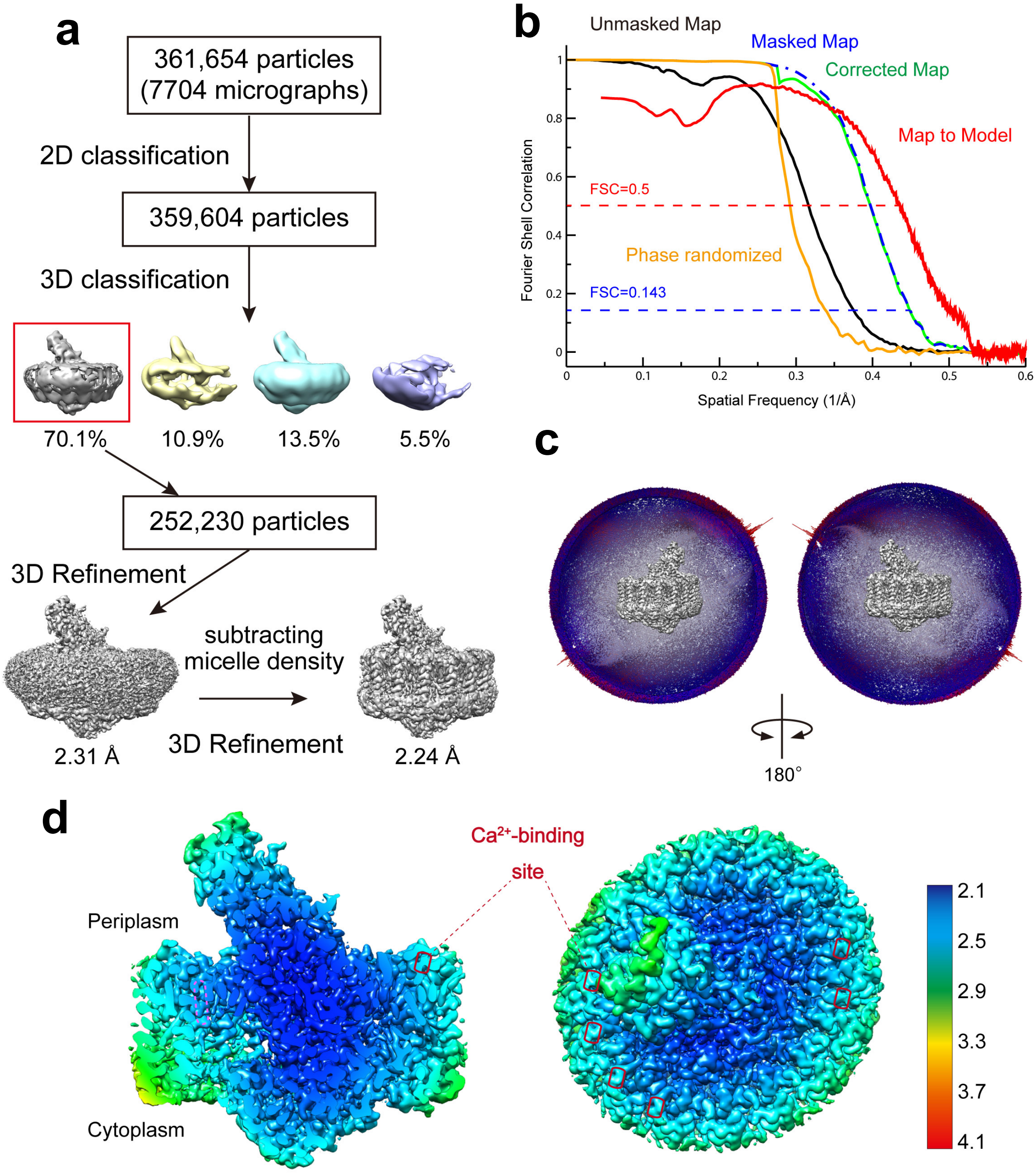

Supplementary Fig. 3 Structure determination of the sucrose-density purified *Alc. vinosum* LH1–RC complex by cryo-EM. (a) Image processing flow of 3D classification and reconstruction. **(b)** The Fourier shell correlation (FSC) plots of the cryo-EM map (unmasked: black, masked: blue, phase randomized corrected: green, phase randomized: orange) and the FSC plot of the model versus the final map (red) are superimposed. **(c)** Angular distribution of reconstructed particles. **(d)** Local resolution representation of the LH1–RC structure. A longitudinal sectional view (*left*) and a top view (*right*) from periplasmic side. The map is shown in rainbow colors as shown in the right color bar.

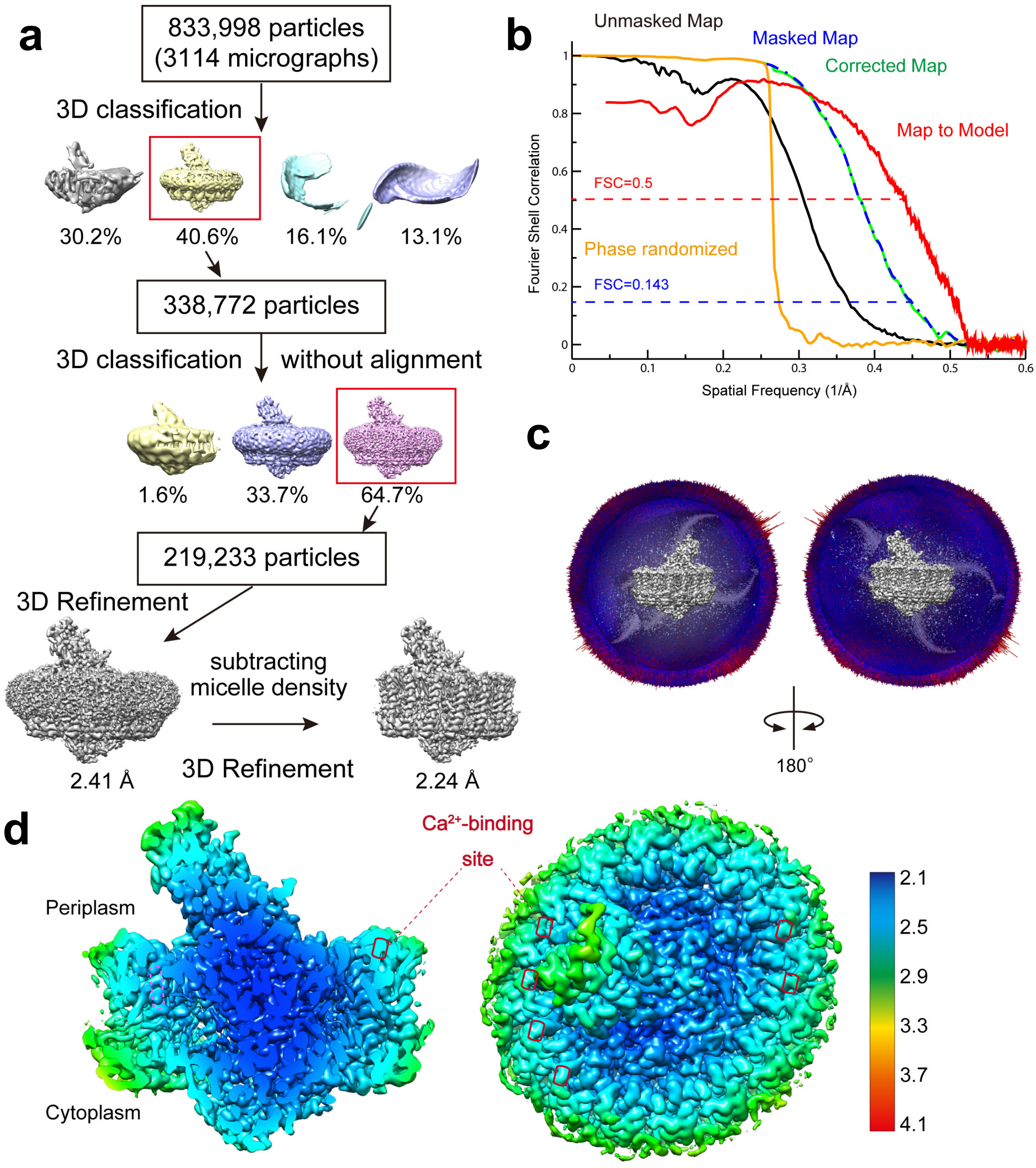

Supplementary Fig. 4 Structure determination of the Ca-DEAE purified *Alc. vinosum* LH1–RC complex by cryo-EM. **(a)** Image processing flow of 3D classification and reconstruction. **(b)** The Fourier shell correlation (FSC) plots of the cryo-EM map (unmasked: black, masked: blue, phase randomized corrected: green, phase randomized: orange) and the FSC plot of the model versus the final map (red) are superimposed. **(c)** Angular distribution of reconstructed particles. **(d)** Local resolution representation of the LH1–RC structure. A longitudinal sectional view (*left*) and a top view (*right*) from periplasmic side. The map is shown in rainbow colors as shown in the right color bar.

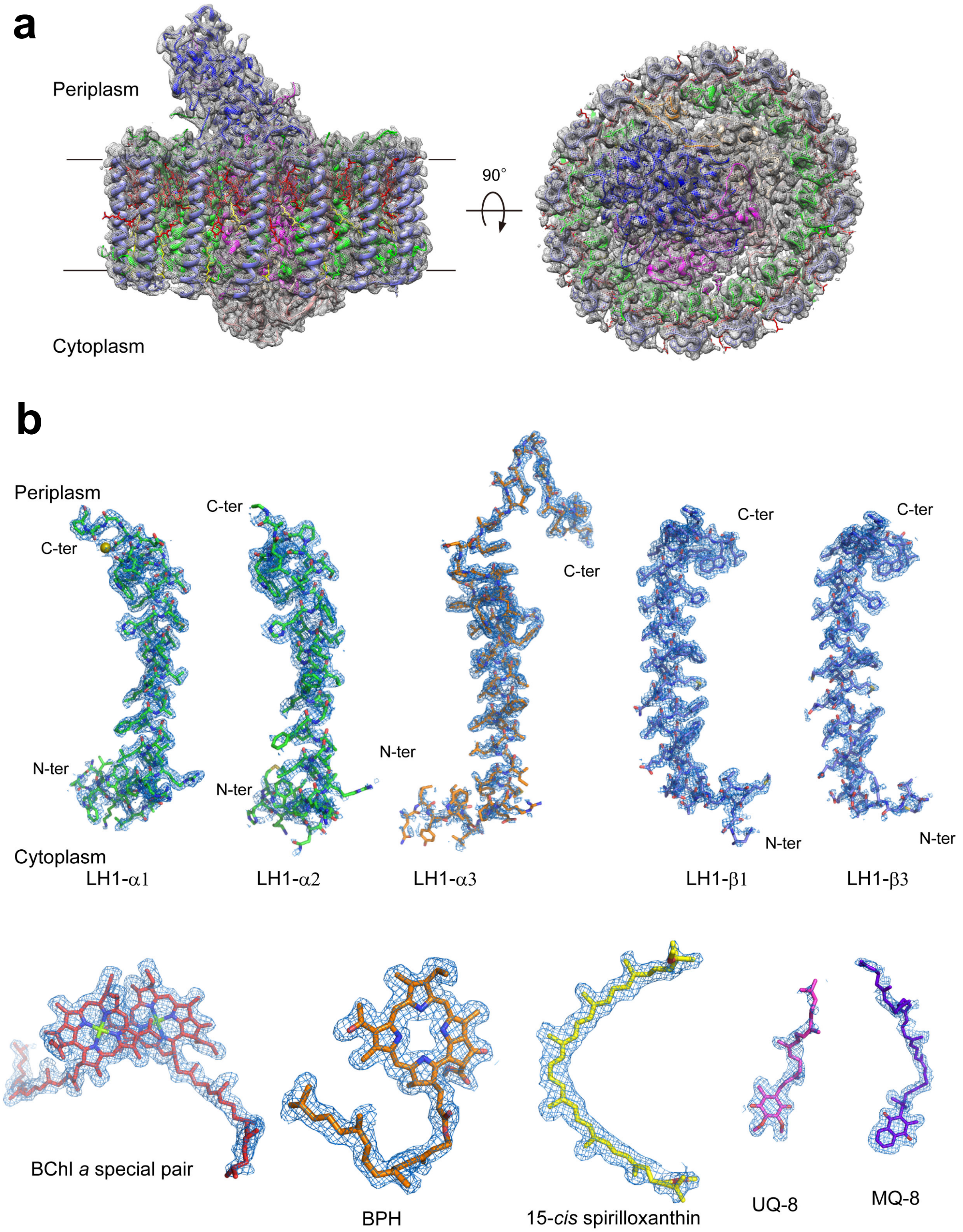

Supplementary Fig. 5 Cryo-EM densities and structural models in the sucrose-density purified *Alc. vinosum* LH1–RC complex. (**a**) Overall structure of the LH1–RC complex. Cartoon representation of the complex with the cryo-EM density in gray mesh. Side view (*left*) parallel to the membrane plane and top view (*right*) from periplasmic side. **(b)** Selected polypeptides and cofactors. The density maps are shown at a contour level of 4.0σ. The color codes are the same as in Fig. 1.

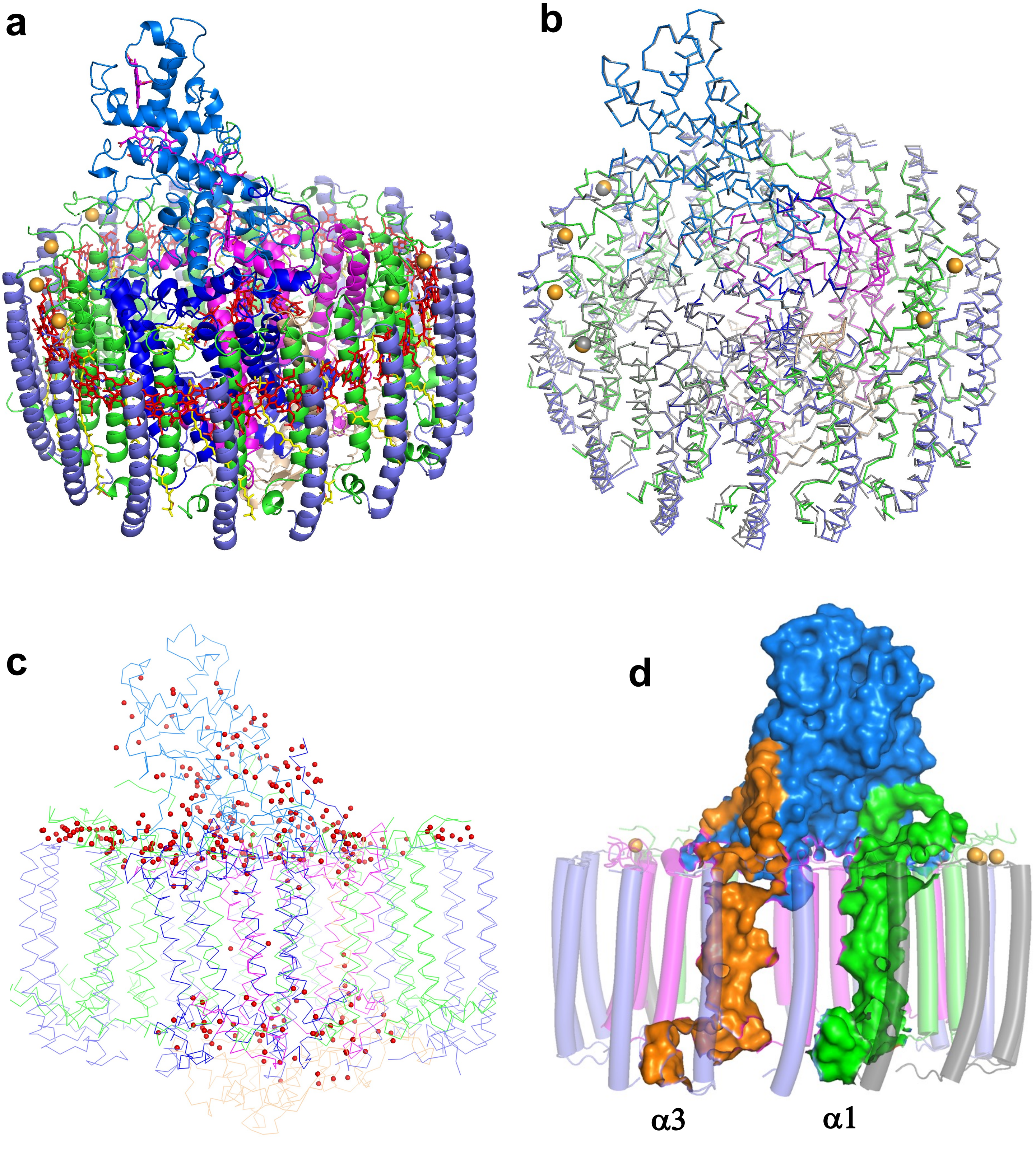

Supplementary Fig. 6 Structure of the *Alc. vinosum* LH1–RC complex. **(a)** Overall structure of the sucrose-density purified LH1–RC complex. **(b)** Overlap view of the *Alc. vinosum* LH1–RC complexes purified using sucrose density gradient centrifugation (colored) and Ca-DEAE chromatography (gray) by superposition of Cα carbons of the RC-M subunits with a root-mean-square deviation (RMSD) of 0.37 Å. **(c)** Side view of the sucrose-density purified LH1–RC complex showing distribution of the bound waters (red balls). **(d)** Interactions between the *Alc. vinosum* LH1 α-polypeptides and the RC C-subunit. The α3-(orange surface) and one of the α1-polypeptides (green surface) interact with the C-subunit (blue surface) through their long C-terminal domains. Only the Ca^{2+} bound to LH1 are represented.

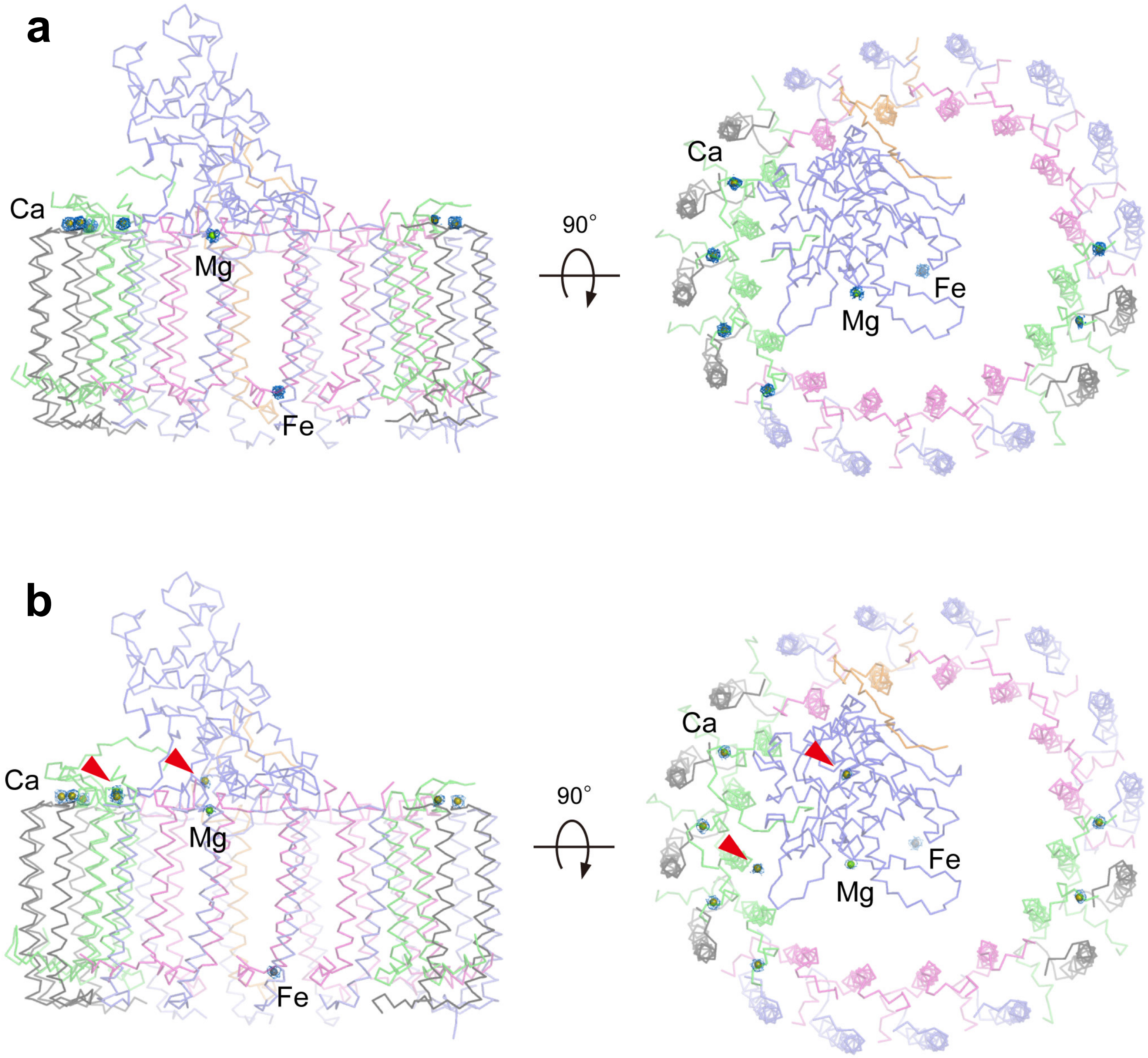

Supplementary Fig. 7 Comparison of the cation binding sites in sucrose-density and Ca-DEAE purified *Alc. vinosum* LH1–RC complexes. (a) Overall structure of the sucrose-density purified LH1–RC complex. **(b)** Overall structure of the Ca-DEAE purified LH1–RC complex. For clarity, L-, M-, H-subunit are removed. Ca, Mg, and Fe ions are shown as spheres in olive, green, gray, respectively. All other color codes are the same as in Fig. 2. Arrow heads indicate additionally found Ca^{2+}-binding sites of the C-subunit in Ca-DEAE purified LH1–RC complex. The density maps corresponding ions are shown as meshes (marine) at a contour level of 4.0σ.

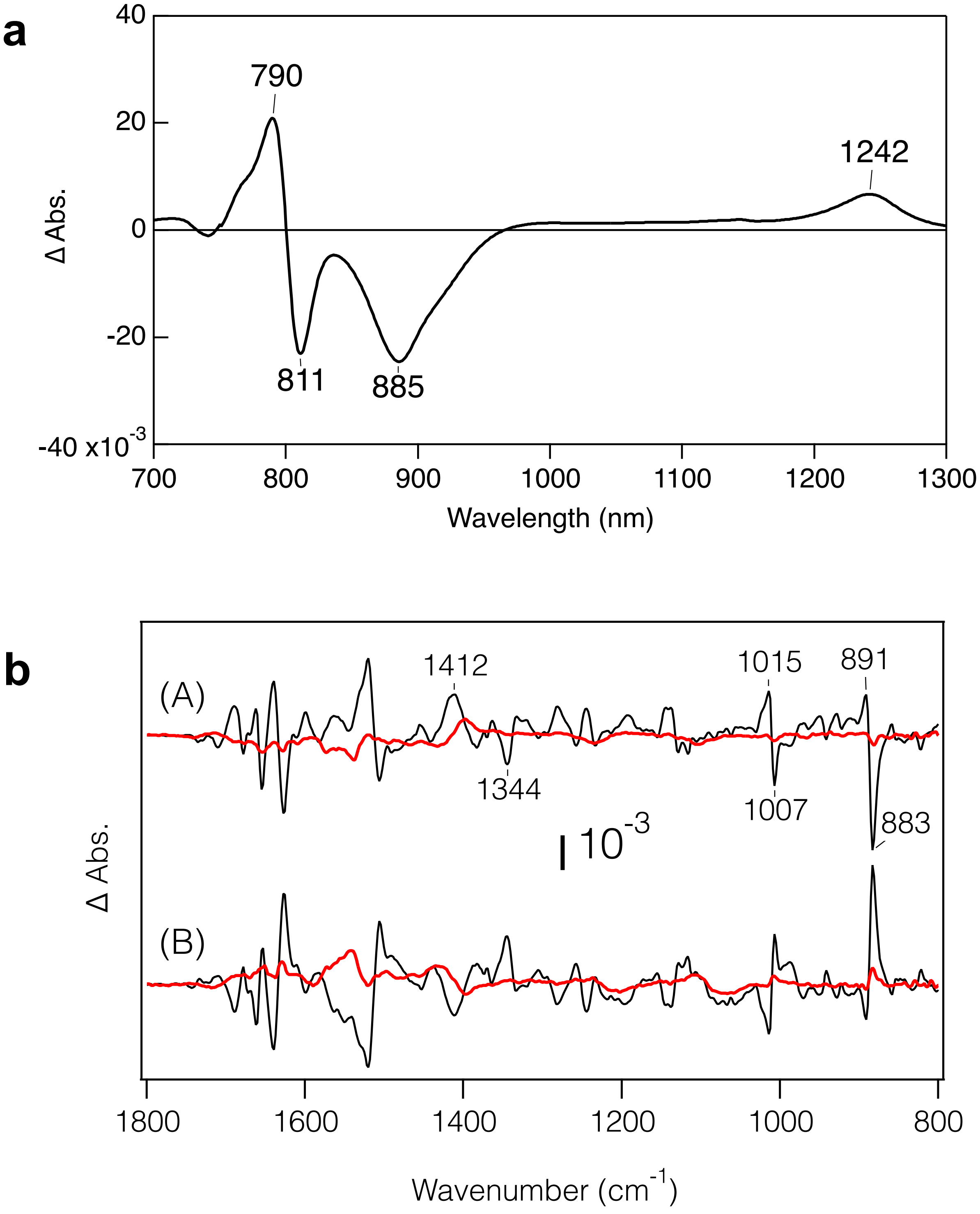

Supplementary Fig. 8 Light-induced absorption spectra and perfusion-induced ATR-FTIR measurements. (a) Light-induced P^+/P absorption difference spectra of the LH1–RC purified from *Alc. vinosum* by subtracting spectrum in the dark from that measured during illumination with 940 nm-LED light. **(b)** ATR-FTIR difference spectra of the LH1–RC complexes from *Alc. vinosum* (red) and *Tch. tepidum* (black) upon Ca^{2+}-to-Sr^{2+} substitution (A) followed by Sr^{2+}-to-Ca^{2+} substitution (B).

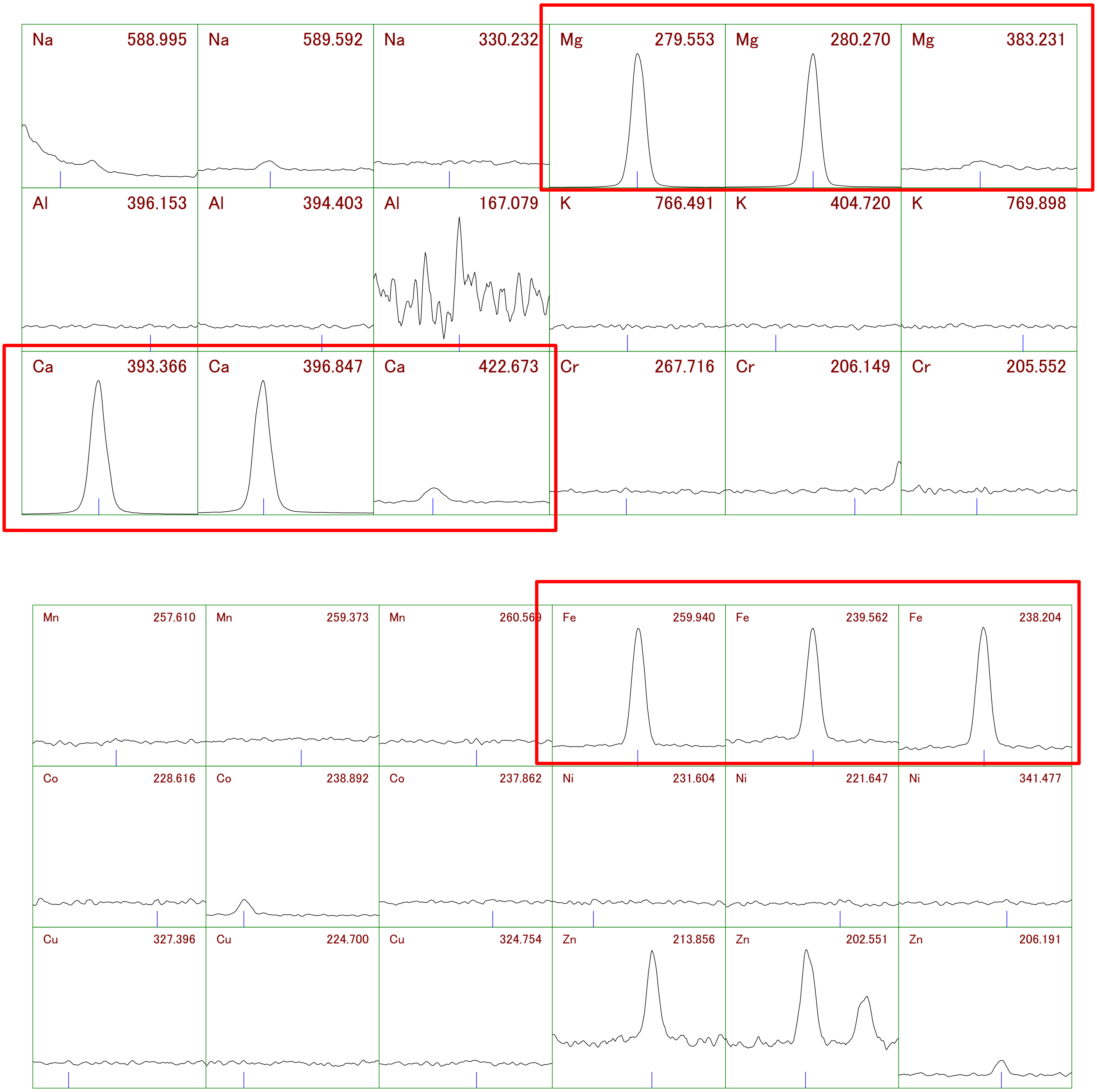

Supplementary Fig. 9 ICP-AES spectra of the sucrose-density purified LH1–RC complex. Each metal ion was detected at three different wavelengths as indicated. Three metal ions (Ca, Mg and Fe) with concentrations above 0.1 ppm are marked by red rectangles. Concentration of LH1–RC was A_{889} = 1.3 cm^{-1} in 20 mM Tris-HCl (pH8.0) buffer containing 0.05% w/v DDM.

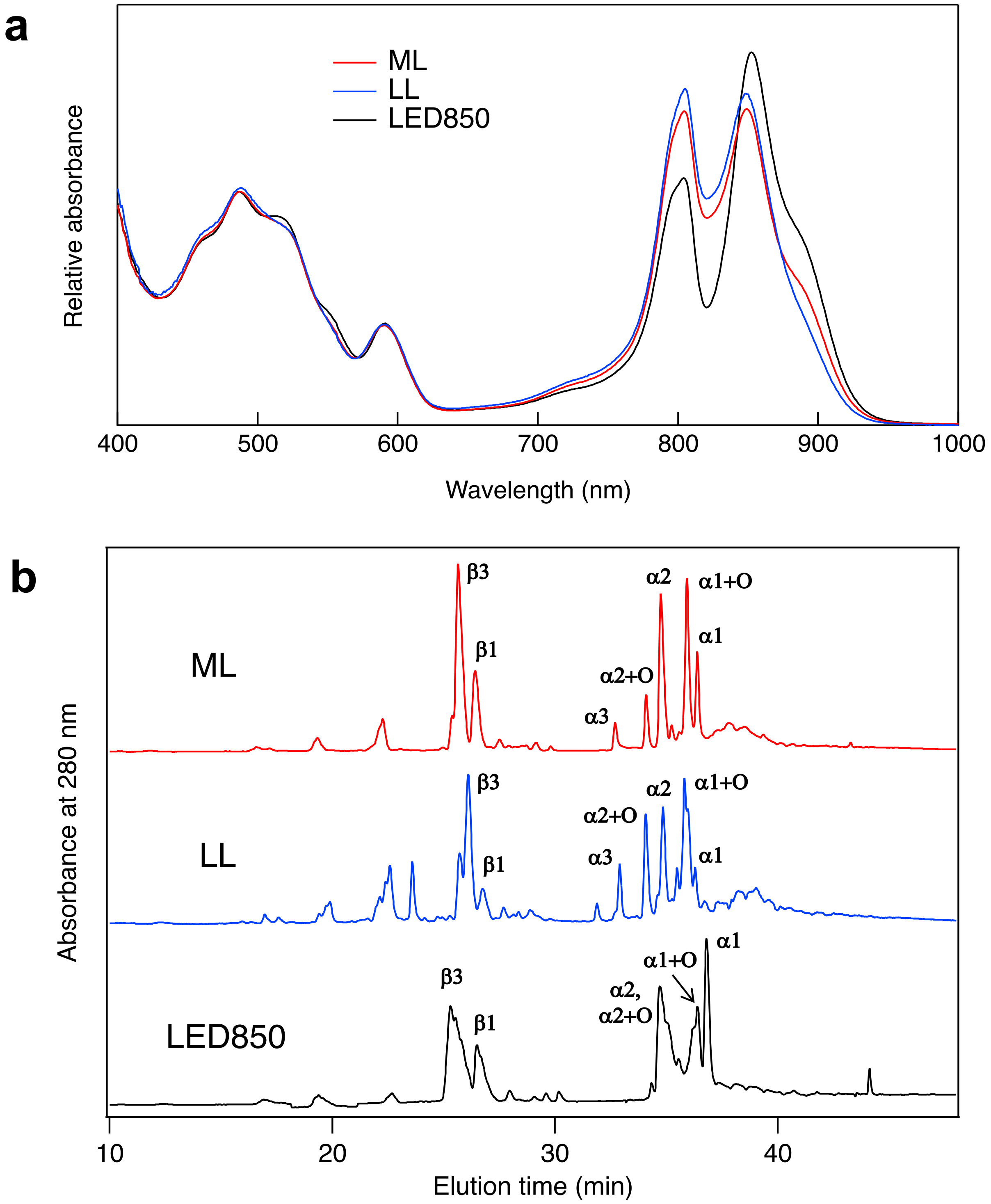

Supplementary Fig. 10 Effects of illumination on the *Alc. vinosum* ICM and LH1 complex. (a) Absorption spectra of ICMs from the cells grown under incandescent illumination at middle light (ML) and low light (LL) intensities and under LED illumination (LED850). **(b)** Reverse-phase HPLC chromatograms (TSKgel, SuperODS, 4.6 × 100 mm, TOSOH) of the LH1 polypeptides purified from the cells grown under different illumination conditions. The polypeptides were eluted at 25°C by a gradient of 40–100% organic solvent consisting of acetonitrile/2-propanol (2:1) containing 0.1% trifluoroacetic acid. Some of the α-polypeptides were oxidized at N-terminal Met residue (+O).

a

```
Alc. vinosum PufA1  MSPDLWKIWLLVDPRRILIAVFAFLTVLGLAIHMILLSTAEFNWLEDGVPAATVQQVTPVVPQR--
Alc. vinosum PufA2  ----MHKIWQIFDPRRTLVALFGFLFVLGLLIHFILLSSPAFNWLSGS------------------
Alc. vinosum PufA3  MMPQLYKIWLAFDPRMALIGLGAFLFALALFIHYMLLRSPEFDWLLGPDYAPVTLSAGMSALPAGR
                        : ***  .***  *:.: .** .*.* ** :** :. *:** .

Alc. vinosum PufB1  --MANSSMTGLTEQEAQEFHGIFVQSMTAFFGIVVIAHILAWLWRPWL
Alc. vinosum PufB2  MANENRSMSGLTEDEAREFHGIFVSSFVVFTGIVVVAHILVWLWRPWL
Alc. vinosum PufB3  -MADQKSMTGLTEEEAKEFHGIFTQSMTMFFGIVIIAHILAWLWRPWL
                        : **:****:**:******..*:. * ***::****.*******
```

b **Sequence alignment scores of *Alc. vinosum* α- and β-polypeptides**

	α2	α3
α1	**59**	41
α2		**55**

	β2	β3
β1	72	**80**
β2		64

c

```
pufA
A.vinosum A1     MSPDLWKIWLLVDPRRILIAVFAFLTVLGLAIHMILLSTAEFNWLEDGVPAATVQQVTPVVPQR
A.tepidum A1     MSPDLWKIWLLIDPRRVLIAVFAFLTILGLAIHMILLSTTEFNWLEDGIPAAKVQQVTPVVPQR

A.vinosum A2     MHKIWQIFDPRRTLVALFGFLFVLGLLIHFILLSSPAFNWLSGS
A.tepidum A2     MHKIWQIFDPRRTLVALFGFLFVLGLLIHFILLSSPAFNWLSGS

A.vinosum A3     MMPQLYKIWLAFDPRMALIGLGAFLFALALFIHYMLLRSPEFDWLLGPDYAPVTLSAGMSALPAGR
A.tepidum A3     MPQQLYKIWLAFDPRMALIGLGAFLFALALFIHYMLLSSPGFDWLLGPDHAPVALSAGMSALPAGR


pufB
A.vinosum B1     MANSSMTGLTEQEAQEFHGIFVQSMTAFFGIVVIAHILAWLWRPWL
A.tepidum B1     MANSSMTGLTEQEAQEFHGIFVQSMTAFFGIVVIAHILAWLWRPWL

A.vinosum B2     MANENRSMSGLTEDEAREFHGIFVSSFVVFTGIVVVAHILVWLWRPWL
A.tepidum B2     MANENRSMSGLTEDEAREFHGIFVSSFVVFTGIVVVAHILVWLWRPWL

A.vinosum B3     MADQKSMTGLTEEEAKEFHGIFTQSMTMFFGIVIIAHILAWLWRPWL
A.tepidum B3     MADQKSMTGLTEDEAKEFHGIFTQSMTMFFGIVIIAHILAWLWRPWL
```

Supplementary Fig. 11 Sequences and sequence comparison of the *Alc. vinosum* LH1 polypeptides. **(a)** Amino acid sequences of α- and β-polypeptides encoded in the *Alc. vinosum pufBA* operons. BChl *a*-coordinating His residues are shown in magenta letters and Ca^{2+}-ligating residues are shown in red letters. Symbol scheme: (*) identical, (.) similar, (:) highly similar. **(b)** Sequence similarities between multiple α- and β-polypeptides using sequence alignment scores from CLUSTAL 2.1. **(c)** Alignments of amino acid sequences for each PufA and PufB pair between *Alc. vinosum* and *Alc. tepidum*. Differences of the residues between the two species are indicated by red letters.

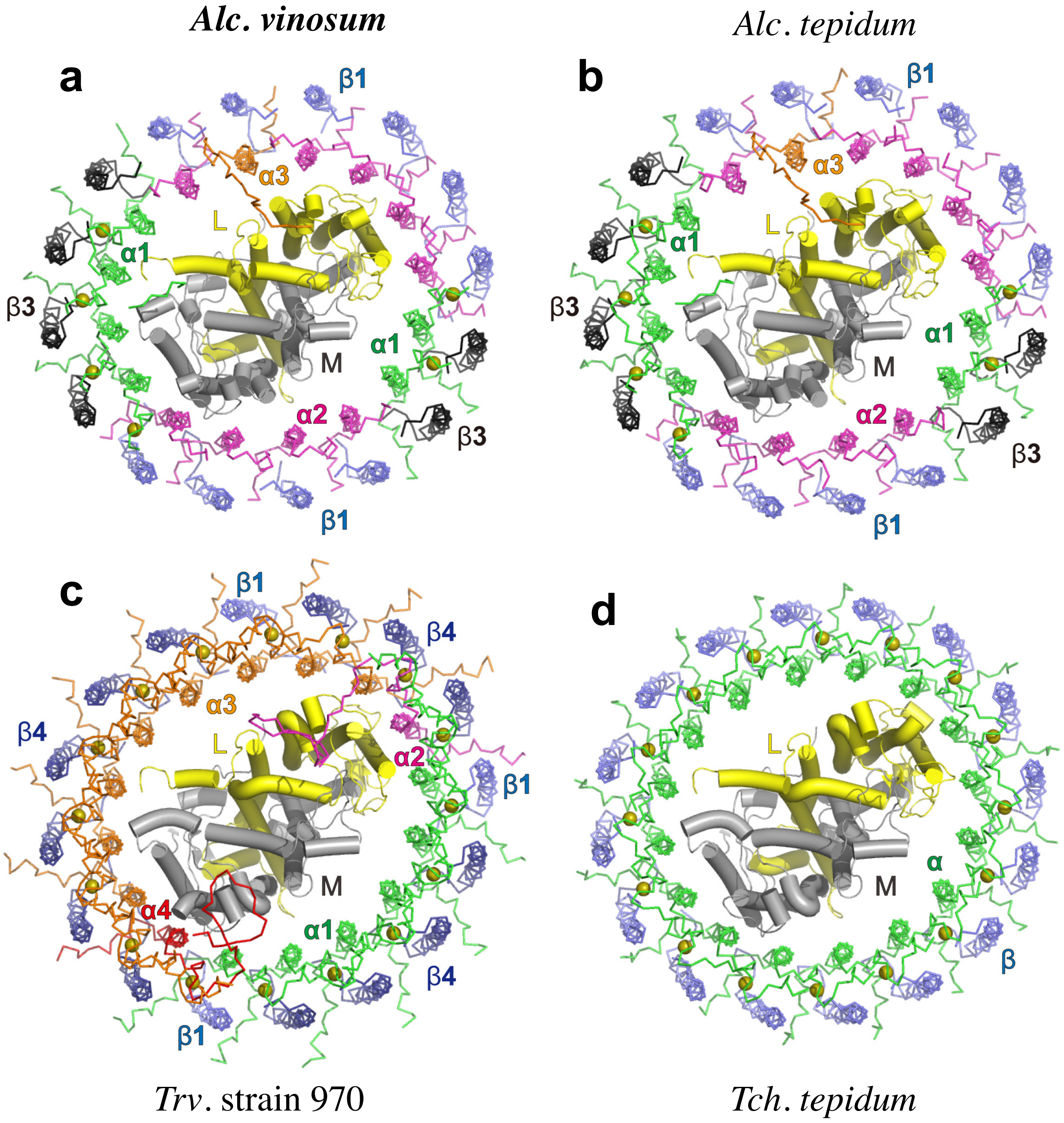

Supplementary Fig. 12 Comparison of arrangement of the *Alc. vinosum* LH1 αβ-polypeptides (a) with those of *Alc. tepidum* (b), *Trv.* strain 970 (c), and *Tch. tepidum* (d). (a) Arrangement of the multiple *Alc. vinosum* LH1 αβ-polypeptides (α1: green, α2: magenta, α3: orange, β1: slate blue, and β3: black) around the RC. Ca ions are shown by olive spheres. **(b)** Arrangement of the multiple *Alc. tepidum* LH1 αβ-polypeptides (α1: green, α2: magenta, α3: orange, β1: slate blue, and β3: black) around the RC. **(c)** Arrangement of the multiple *Trv.* strain 970 LH1 αβ-polypeptides (α1: green, α2: magenta, α3: orange, α4: red, β1: slate blue, and β4: blue) around the RC. **(d)** Arrangement of the LH1 αβ-polypeptides (α: green and β: slate blue) from *Tch. tepidum* around the RC, L-subunit (yellow) and M-subunit (gray).

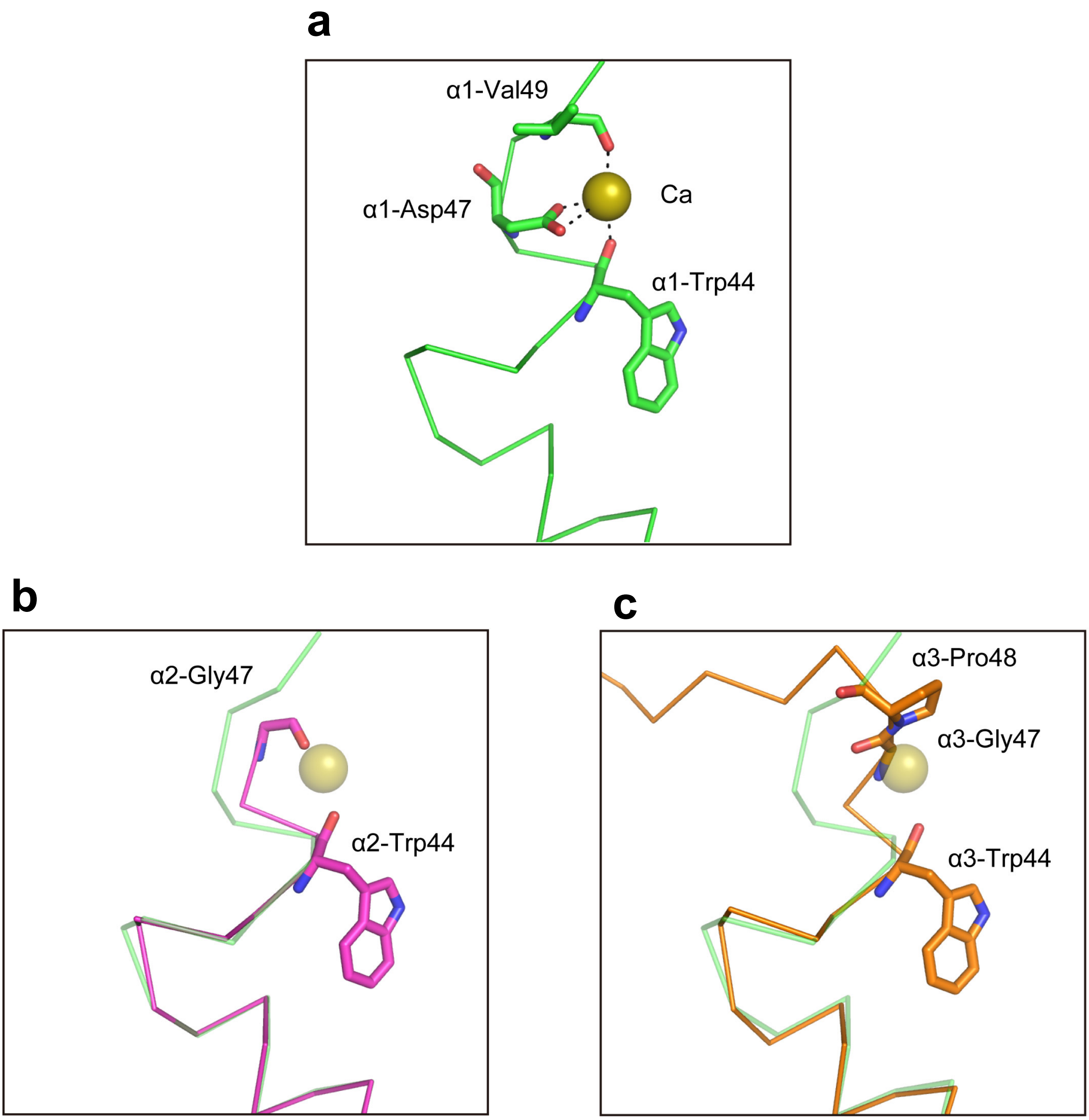

Supplementary Fig. 13 Comparison of α-polypeptides around Ca^{2+}-binding sites in the *Alc. vinosum* LH1 complex. **(a)** A typical Ca^{2+}-binding site in α1-polypeptide (green). Dashed lines indicate hydrogen bonds or salt bridges within a distance of 3.0 Å. **(b)** Superposition of Cα carbons of the α1-polypeptide (transparent color) with that of α2-polypeptide (magenta) showing the disruption of the Ca^{2+} binding site in C-terminus of the α2-polypeptide. **(c)** Superposition of Cα carbons of the α1-polypeptide (transparent color) with that of α3-polypeptide (orange) showing the different conformations around the Ca^{2+}-binding site. Ca^{2+} is shown as an olive sphere.

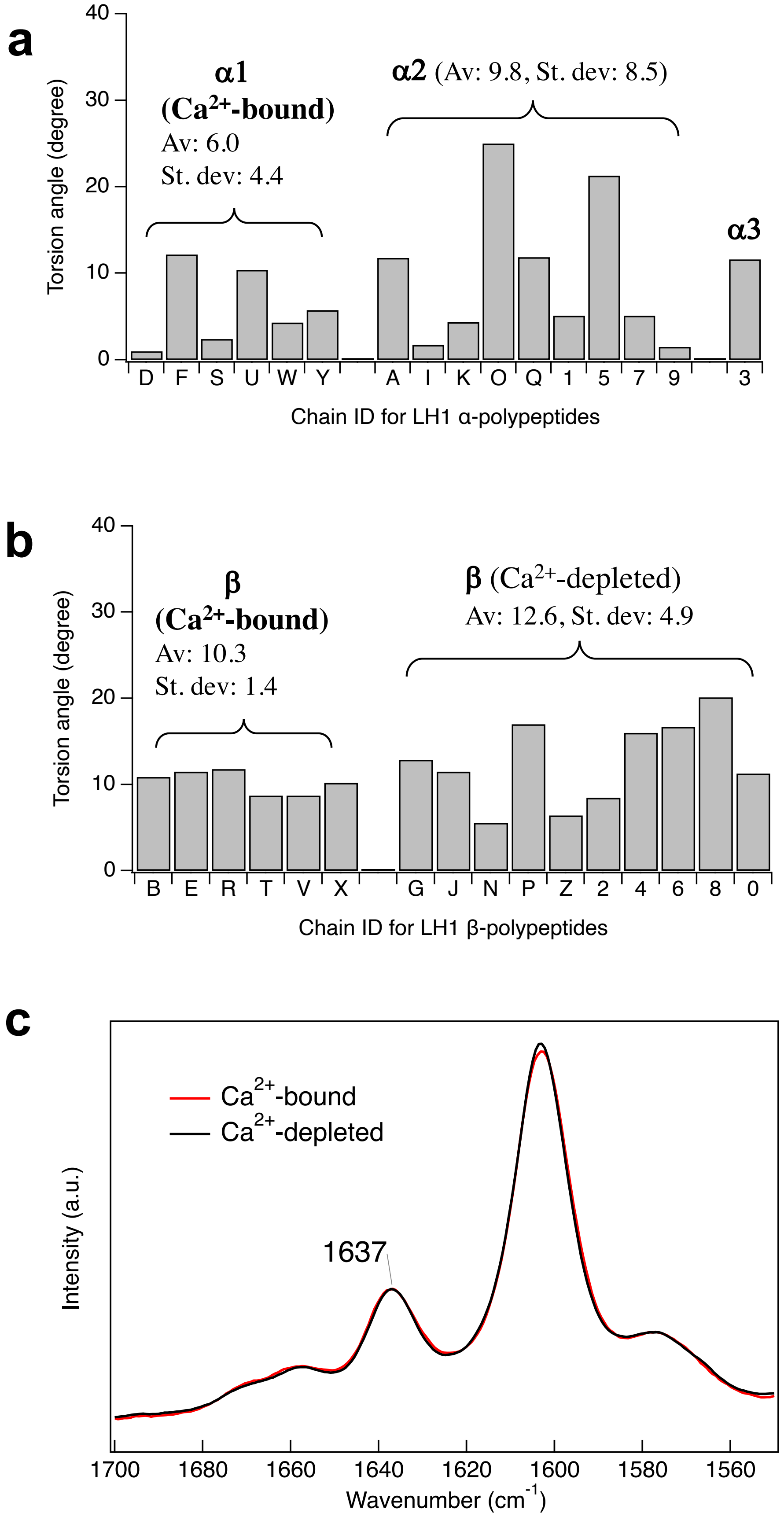

Supplementary Fig. 14 Torsion angle distributions and resonance Raman spectra of the BChl *a* C3-acetyl group in *Alc. vinosum* LH1. (a) Torsion angle distribution for the BChls *a* in α-polypeptides. **(b)** Torsion angle distribution for the BChls *a* in β-polypeptides. **(c)** Resonance Raman bands (355-nm excited) of the BChl *a* C3-acetyl group in Ca^{2+}-bound and Ca^{2+}-depleted LH1–RCs.

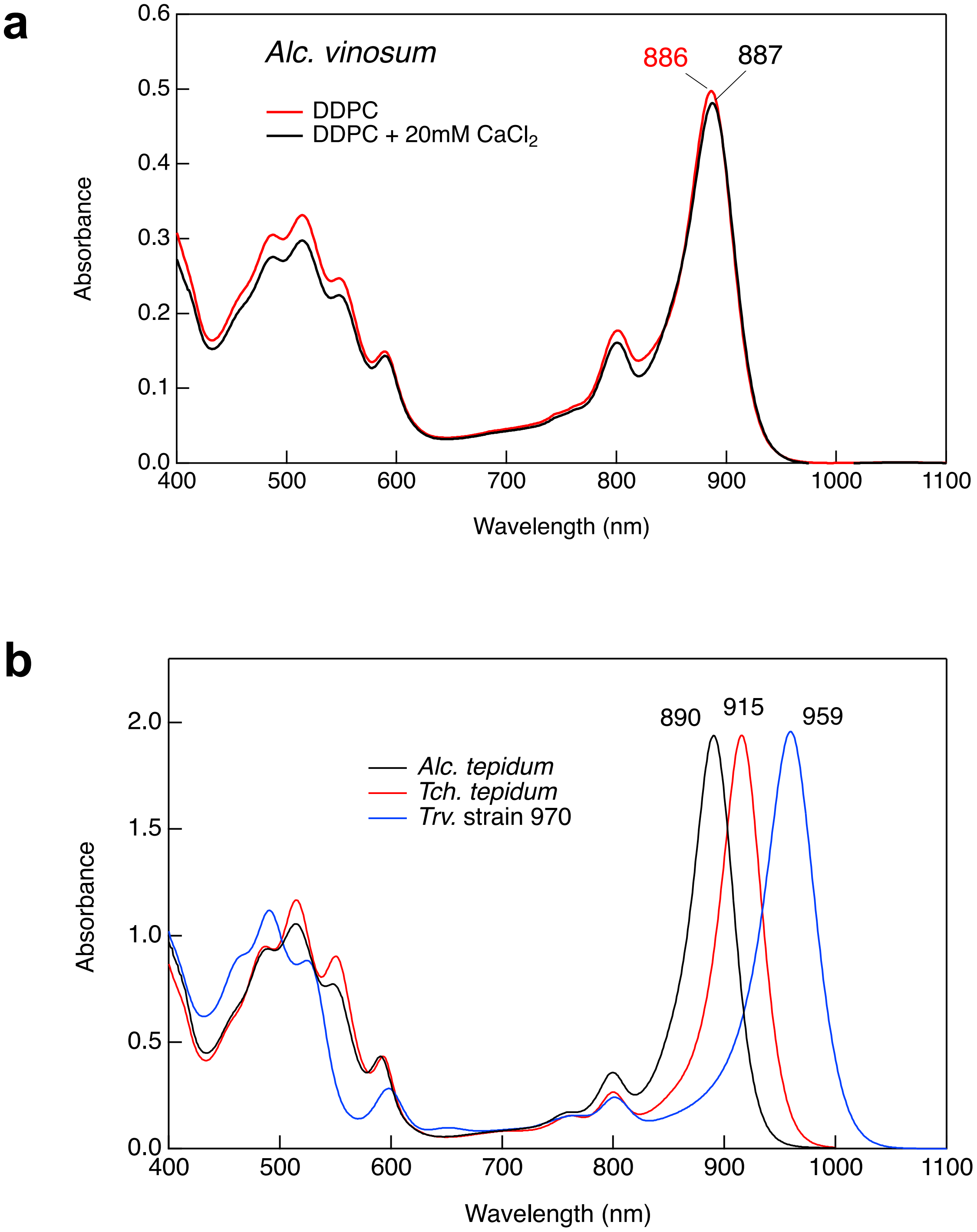

Supplementary Fig. 15 Effects of detergent on the absorption spectra of LH1–RC complexes. (a) Absorption spectra of the *Alc. vinosum* LH1–RC complex purified by sucrose density gradient centrifugation using DDPC (red curve) followed by addition of 20 mM $CaCl_2$ (black curve). **(b)** Absorption spectra of the LH1–RC complexes from *Alc. tepidum*, *Tch. tepidum* and *Trv.* strain 970 purified using DDM.

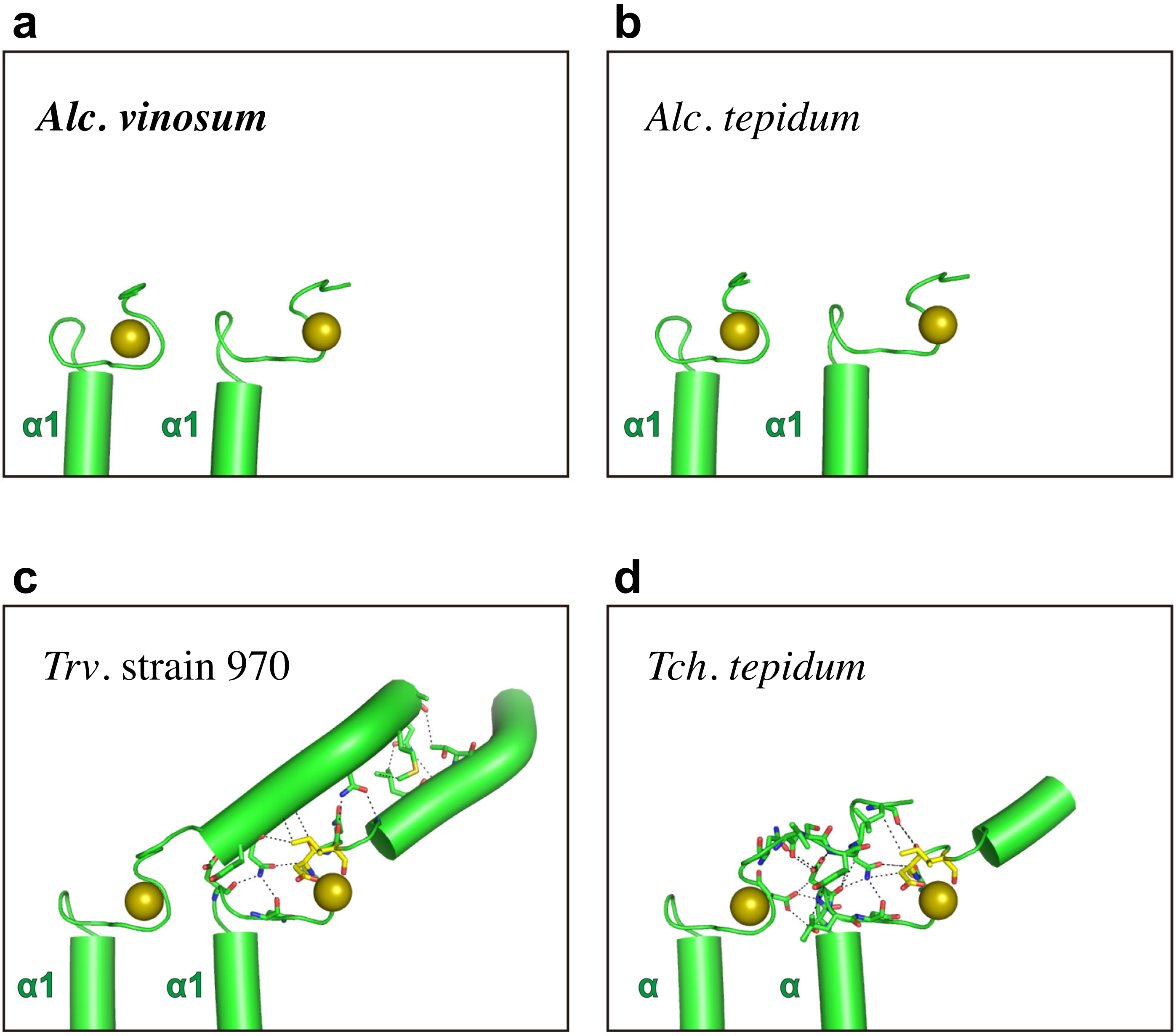

Supplementary Fig. 16 Interaction manner between C-terminal domains following the Ca^{2+}-binding site in LH1 α-polypeptides. (a) Due to disorder at the C-terminus, no interaction was found between C-terminal domains of LH1 α1-polypeptides of *Alc. vinosum*. **(b)** Due to disorder at the C-terminus, no interaction was found between C-terminal domains of LH1 α1-polypeptides of *Alc. tepidum*. **(c)** Parallel helical interaction between C-terminal domains of LH1 α1-polypeptides of *Trv.* strain 970. **(d)** Loop-loop interaction between C-terminal domains of LH1 α-polypeptides of *Tch. tepidum*. All interacting residues (<4.0 Å) are shown in green sticks, and further bound to Ca^{2+} are shown in yellow sticks. Ca ions are shown as spheres in olive.
