# Supplementary material for: High-resolution structure and biochemical properties of the LH1–RC photocomplex from the model purple sulfur bacterium, Allochromatium vinosum
Source: Commun Biol. 2024 Feb 12;7:176. doi: 10.1038/s42003-024-05863-w (PMC10861460; doi:10.1038/s42003-024-05863-w)
Supplement: Supplementary file 3 — Description of Additional Supplementary Files [file 42003_2024_5863_MOESM3_ESM.pdf]

Description of Additional Supplementary Files

File name: Supplementary Data 1
Description: The numerical source values underlying Figure 4.
